# Bigger problems from smaller colonies: emergence of antibiotic-tolerant small colony variants of *Mycobacterium avium* complex in MAC-pulmonary disease patients

**DOI:** 10.1186/s12941-024-00683-6

**Published:** 2024-03-18

**Authors:** Hyun-Eui Park, Kyu-Min Kim, Minh Phuong Trinh, Jung-Wan Yoo, Sung Jae Shin, Min-Kyoung Shin

**Affiliations:** 1 Department of Microbiology and Convergence of Medical Science, College of Medicine, Gyeongsang National University, Jinju, 52727 Republic of Korea; 2 Department of Internal Medicine, Gyeongsang National University Hospital, Jinju, 52727 Republic of Korea; 3 Department of Microbiology, Institute for Immunology and Immunological Diseases, Brain Korea 21 Program for Leading Universities and Students (PLUS) Project for Medical Science, Yonsei University College of Medicine, Seoul, 03722 Republic of Korea

**Keywords:** Non-tuberculous mycobacteria, Mycobacterium avium complex, Small colony variant, Revertant colony, Antibiotic-tolerance

## Abstract

**Background:**

*Mycobacterium avium* complex (MAC) is a group of slow-growing mycobacteria that includes *Mycobacterium avium* and *Mycobacterium intracellulare*. MAC pulmonary disease (MAC-PD) poses a threat to immunocompromised individuals and those with structural pulmonary diseases worldwide. The standard treatment regimen for MAC-PD includes a macrolide in combination with rifampicin and ethambutol. However, the treatment failure and disease recurrence rates after successful treatment remain high.

**Results:**

In the present study, we investigated the unique characteristics of small colony variants (SCVs) isolated from patients with MAC-PD. Furthermore, revertant (RVT) phenotype, emerged from the SCVs after prolonged incubation on 7H10 agar. We observed that SCVs exhibited slower growth rates than wild-type (WT) strains but had higher minimum inhibitory concentrations (MICs) against multiple antibiotics. However, some antibiotics showed low MICs for the WT, SCVs, and RVT phenotypes. Additionally, the genotypes were identical among SCVs, WT, and RVT. Based on the MIC data, we conducted time-kill kinetic experiments using various antibiotic combinations. The response to antibiotics varied among the phenotypes, with RVT being the most susceptible, WT showing intermediate susceptibility, and SCVs displaying the lowest susceptibility.

**Conclusions:**

In conclusion, the emergence of the SCVs phenotype represents a survival strategy adopted by MAC to adapt to hostile environments and persist during infection within the host. Additionally, combining the current drugs in the treatment regimen with additional drugs that promote the conversion of SCVs to RVT may offer a promising strategy to improve the clinical outcomes of patients with refractory MAC-PD.

## Introduction

Non-tuberculous mycobacteria (NTM) are environmental mycobacteria other than the *Mycobacterium tuberculosis* complex and *Mycobacterium leprae* [1]. NTM are ubiquitous not only in natural environments, such as soil and water, but also in household environments, such as showerheads, kitchen sinks, plumbing, and refrigerator taps [2]. Although most NTM are environmental saprophytes, several species are pathogenic and cause infections in various parts of the human body, such as the lungs, lymph nodes, bones, and soft tissues [3]. Among these disease types, NTM-pulmonary disease (PD) is the most common clinical presentation comprising approximately 90% of NTM infections [1].

*Mycobacterium avium* complex (MAC), which comprises multiple species, such as *Mycobacterium avium*, *Mycobacterium intracellulare*, *Mycobacterium chimaera*, and other minor species, is the most common cause of NTM-PD worldwide [4, 5]. MAC pulmonary infection is an emerging pulmonary disease, particularly in immunocompromised patients or immunocompetent patients with chronic pulmonary diseases such as bronchiectasis, chronic obstructive pulmonary disease, cystic fibrosis, emphysema, and previous pulmonary tuberculosis [6, 7]. However, the treatment of MAC-PD is challenging due to the emergence of antibiotic resistance and toxic adverse effects related to prolonged antibiotic treatment [8]. Previous studies demonstrated that 20 to 40% of patients do not respond to the three-drug regimen including macrolide, ethambutol, and rifampicin [9–11]. Furthermore, clinical recurrence is commonly observed in a considerable proportion of successfully treated patients [7, 12–14].

A broad range of bacterial pathogens, such as *Staphylococcus aureus* and *Pseudomonas aeruginosa* survive within the host by altering their phenotype to a small colony variant (SCV) type [15–19]. SCV is a bacterial subpopulation with unique microbiological features such as reduced colony size, decreased pigmentation, slow growth rate, and increased antibiotic resistance [20]. Owing to its unique characteristics, such as slow metabolic rates and resistance to multiple antibiotics, SCV has emerged as a significant challenge in curing chronic or persistent infections [16, 18, 20]. Therefore, dissecting the biology of SCV is essential for effective treatment of infectious disease. However, SCV have not been well recognized in clinical microbiology laboratories for several reasons. Owing to slow growth rate of SCV, it is overshadowed by the wild-type (WT) strain with a fast growth rate during cultivation. In addition, the shape and size of the colonies are highly different from those of the wild-type: therefore, they can be mistaken for contaminating bacteria. Furthermore, SCV may return to its wild-type form after a certain period or when inoculated into nutrient- rich media [21].

Various stress conditions are known to induce SCV formation in vitro conditions. Besse et al. revealed that the redox imbalance caused by O_2_ limitation cause SCV formation in *Pseudomonas aeruginosa* [16]. In addition, eliminating O_2_ limitation by aeration or alternative electron acceptors significantly reduces the proportion of the SCV phenotype [16]. Similarly, previous studies have shown that antibiotic treatment induces SCV formation. Fauerharmel-Nunes et al. demonstrated that prolonged vancomycin exposure facilitated the generation of nonstable SCV of *S. aureus* in a macrophage and endothelial cell infection model [18]. In addition, streptomycin treatment induced the emergence of the SCV phenotype in *Salmonella Typhimurium* by a frameshift mutation in *ubiE*, consequently promoting biofilm formation [22]. Few studies have been conducted on SCV from NTM diseases [23–25]. Previous studies have reported that MAC has two forms: translucent and opaque colony [23–25]. Primary cultures from patients with NTM lung disease often show mixed translucent and opaque phenotypes, and these two colony types are reversible [23, 24]. However, we still lack comprehensive knowledge regarding the specific traits of SCVs in MAC; their contribution to the development of MAC-lung disease remains largely unknown, and their role in the pathogenesis of MAC lung disease still needs to be elucidated. Therefore, we investigated the phenotypic and genotypic characteristics of SCV isolated from respiratory specimens of patients with MAC lung disease.

## Methods

### *Mycobacterium avium* complex isolates

A total of 107 MAC isolates were collected from respiratory specimens of NTM-suspected patients admitted to the Gyeongsang National University Hospital (GNUH, Jinju, Korea) between 2017 and 2022. Bacterial resources and related clinical information were provided by the Fastidious Specialized Pathogen Resources Bank (a constituent of the National Culture Collection for Pathogens, GNUH), Jinju, Korea. Clinical information contains the clinical characteristics of the patients, including the age, gender, comorbidity, treatment initiation, and isolated species. All MAC isolates were inoculated into 7H10 agar and incubated at 37℃ for 2–4 weeks until colonies became visible. After incubation, all small/translucent and large/opaque colonies were selected for further identification. All selected isolates were identified using the multiplex PCR assay described in our previous study [26] and stored at -80℃ for further experiments.

Nine MAC isolates were randomly selected from 107 isolates for further analysis. These isolates consisted of three *M. intracellulare* (MI #28, #410, and #5151), three *M. avium* subsp. *hominissuis* (MAH #229, #460, and #634), and three *M. avium* subsp. *avium* (MAA #273, #3111, and #476). All selected MAC isolates had heterogeneous phenotypes, including WT, SCV, and revertant (RVT). The primary stock of MAC isolates was inoculated into 7H10 agar and 7H10-CRE agar (containing clarithromycin at 2 µg/ml, rifampicin at 1 µg/ml, and ethambutol at 2 µg/ml) and incubated at 37 °C for 3 weeks. The morphological characteristics of the colonies and the presence of SCVs were observed and recorded. Furthermore, the size of the colonies was measured after 4 weeks of incubation at 37℃ in 7H10 agar. A total of 15 colonies were randomly selected, and the size of the colonies was measured using the cellSens standard software (Olympus Life Science).

### Growth characteristics

A single colony of pure MAC cultures including WT, SCVs, and RVT was transferred to 10 ml of Middlebrook 7H9 broth supplemented with 10% OADC and 0.5% glycerol (MB7H9 broth). Inoculated cultures were incubated for 30 days at 37 °C with 250 rpm. During incubation, the bacterial growth was evaluated using a Multiskan SkyHigh microplate spectrophotometer (Thermo Fisher Scientific, Waltham, MA, USA) by measuring the optical density at 600 nm (OD_600_) of the culture. OD_600_ values were measured every 3 days with three biological replicates.

### Acid-fast staining

Loopfuls of pure MAC cultures were suspended in 500 µL of PBS and mixed thoroughly by vortex mixer. After that, 10 µL of bacterial suspension was dropped onto slide glass and dried thoroughly. The samples were heat-fixed. Subsequently, the carbolfuchsin reagent was added to the slide and allowed to stain for 30 min. The samples were then washed indirectly with deionized water. Subsequently, acid alcohol was added dropwise for 15 s for decolorization. For counter-staining, we added the methylene blue and allowed to stain for 1 min. Finally, the samples were rinsed with distilled water and air-dried. The stained samples were examined for the presence of acid-fast bacteria under oil-immersion (1000x) using a light microscope.

### Antimicrobial susceptibility testing

Antimicrobial susceptibility to 18 antimicrobial agents, namely amikacin (AMK), streptomycin (STR), rifampicin (RFP), rifabutin (RFB), ethambutol (ETB), clarithromycin (CLR), fidaxomicin (FDX), moxifloxacin (MXF), clofazimine (CFZ), macozinone (MCZ), bedaquilline (BDQ), prothionamide (PTM), linezolid (LZD), tedizolid (TZD), SQ109, tigecycline (TGC), isoniazid (INZ), and epetraborole (EPB), was evaluated by broth microdilution method following the Clinical and Laboratory Standards Institute (CLSI) guidelines with slight modifications [27]. In brief, a single colony of pure MAC culture was inoculated to 5 ml of MB7H9 broth and incubated for 2–3 weeks at 37 °C with 250 rpm. Subsequently, the cultures were diluted to an OD_600_ = 0.13. The diluted bacterial suspension was resuspended to MB7H9 broth at a ratio of 1:100. Following this, 100 µL of prepared bacterial suspension was inoculated to antibiotic panels and incubated at 37 °C in ambient air. Visual inspection was performed at 14 days after incubation to determine the minimum inhibitory concentration (MIC) values. Antimicrobial susceptibility testing results were interpreted as susceptible (S), intermediate (I), or resistant (R), according to CLSI breakpoints and previous studies [27–29].

### Molecular genotyping of MAC isolates

DNA was prepared by heating method. Briefly, a loopful of pure MAC cultures from Middlebrook 7H10 agar was suspended in 200 µL of distilled water and incubated at 95 ℃ for 15 min in a heating block. The frozen samples were centrifuged for 5 min at 13,000 rpm, and the supernatant was used as the DNA template. For genotyping, variable-number tandem-repeat (VNTR) PCR was performed as described previously [30, 31]. In brief, PCR mixtures were prepared including DNA template (3 µL), AccuPower® PCR PreMix (Bioneer, South Korea), forward and reverse primer sets (both 10 µM), and nuclease-free water (Invitrogen, USA) to a final volume of 20 µL. For *M. intracellulare*, the VNTR PCR parameters were as follows: one cycle of 95 °C for 10 min, followed by 38 cycles of 94 °C for 30 s, 60 °C for 30 s, 72 °C for 60 s, and a final extension of 72 °C for 7 min. For *M. avium*, the PCR conditions were as follows: one cycle of 95 °C for 10 min followed by 38 cycles of 98 °C for 10 s, 68 °C for 30 s, and 72 °C for 1 min and a final extension of 72 °C for 7 min. The amplified PCR products were confirmed by gel electrophoresis on 2% agarose gels and visualized using UV transilluminators.

#### Time-kill kinetics of three antimicrobial drug combinations against *M. avium* complex

We tested three drug combinations targeting all MAC phenotypes to investigate their bactericidal effects. The WT, SCVs, and RVT strains of MI #5151 and MAH #460 were cultured on 7H10 agar and resuspended in PBS at an OD_600_ of 0.1. Subsequently, 100 µL of the bacterial suspension was inoculated into 5 ml of MB7H9 broth containing three different antimicrobial drug combinations. These combinations included the standard regimen drugs (CRE, containing CLR at 2 µg/ml, RFP at 1 µg/ml, and ETB at 2 µg/ml), the CB combination (containing CLR at 2 µg/ml and BDQ at 0.25 µg/ml), and the CP combination (CLR at 2 µg/ml and PTM at 32 µg/ml). BDQ and PTM were selected for drug combinations because they showed low MIC values against all MAC phenotypes, including SCVs. Samples were incubated in a shaking incubator at 37 °C and 250 rpm. The bacterial survival of each strain was assessed by CFU-based counting on days 0, 3, and 7 after initial inoculation.

### Statistical analysis

Descriptive statistics were used to investigate the statistical significance of the data for the categorical variables of patients, growth curves, antimicrobial susceptibility testing, colony size measurement, and time-kill kinetics. Data were analyzed using the Kruskal-Wallis test for MIC values and ANOVA for colony size measurement and time-kill kinetics using GraphPad Prism (version 8.0). Furthermore, clinical characteristics of patients according to the presence of SCV were analyzed with the unpaired Student’s *t*-test (for age) and chi-squared test (for the rest of the variables) using Jamovi (version 2.4.11). The *p*-value < 0.05 was considered statistically significant. All data were expressed as mean values ± standard deviations.

## Results

### Clinical characteristics of patients

A total of 107 patients were included in this study. The mean age of patients with and without SCV was 75.8 and 74.2, respectively. Moreover, the proportions of female gender in with and without SCV groups were 34 and 50.9%, respectively. The most prevalent underlying disease was chronic obstructive pulmonary disease, followed by bronchiectasis and pneumonia, according to the description in Table 1. None of the variables showed any significant difference between the patients with and without SCV. The proportion of treatment initiations was similar between patients with and without SCV. *Mycobacterium intracellulare* (MI) is the most common etiological agent, followed by *Mycobacterium avium* subsp. *avium* (MAA), and *Mycobacterium avium* subsp. *hominissius* (MAH).

**Table 1:** Clinical characteristics of the 107 patients with *Mycobacterium avium* complex pulmonary disease, classified by the presence of small colony variants

Characteristic		Total (*n* = 107)	Patients with coexistence of wild-type and small colony variants (*n* = 50)	Patients with wild-type only (*n* = 57)	P-value
Age (years), mean ± S.D.		74.9 ± 10.9	75.8 ± 9.7	74.2 ± 9.7	0.429
Female gender, n (%)		46 (43)	17 (34)	29 (51)	0.079
Male gender, n (%)		61 (57)	33 (66)	28 (49)	
Comorbidity, n (%)					
	Previous history of tuberculosis	5 (4.7)	3 (6)	2 (3.5)	0.869
	Bronchiectasis	10 (9.3)	6 (12)	4 (7)	0.377
	Chronic obstructive pulmonary disease	20 (18.7)	11 (22)	9 (15.8)	0.563
	Asthma	5 (4.7)	2 (4)	3 (5.3)	0.832
	Malignancy	4 (3.7)	1 (2)	3 (5.3)	0.498
	Pneumonia	10 (9.3)	7 (14)	3 (5.3)	0.411
	Bronchiolitis	7 (6.5)	2 (4)	5 (8.8)	0.319
	Hemoptysis	8 (7.5)	5 (10)	3 (5.3)	0.353
Treatment initiation, n (%)		36 (33.6)	16 (32)	20 (35)	0.922
Isolated species, n (%)					0.222
	*Mycobacterium intracellulare*	70 (65.4)	31 (62)	39 (68.4)	
	*Mycobacterium avium* subsp. *hominissius*	14 (13.1)	9 (18)	5 (8.8)	
	*Mycobacterium avium* subsp. *avium*	23 (21.5)	10 (20)	13 (22.8)	

### Phenotypic characteristics of MAC WT, SCVs, and RVT

**Fig. 1:**
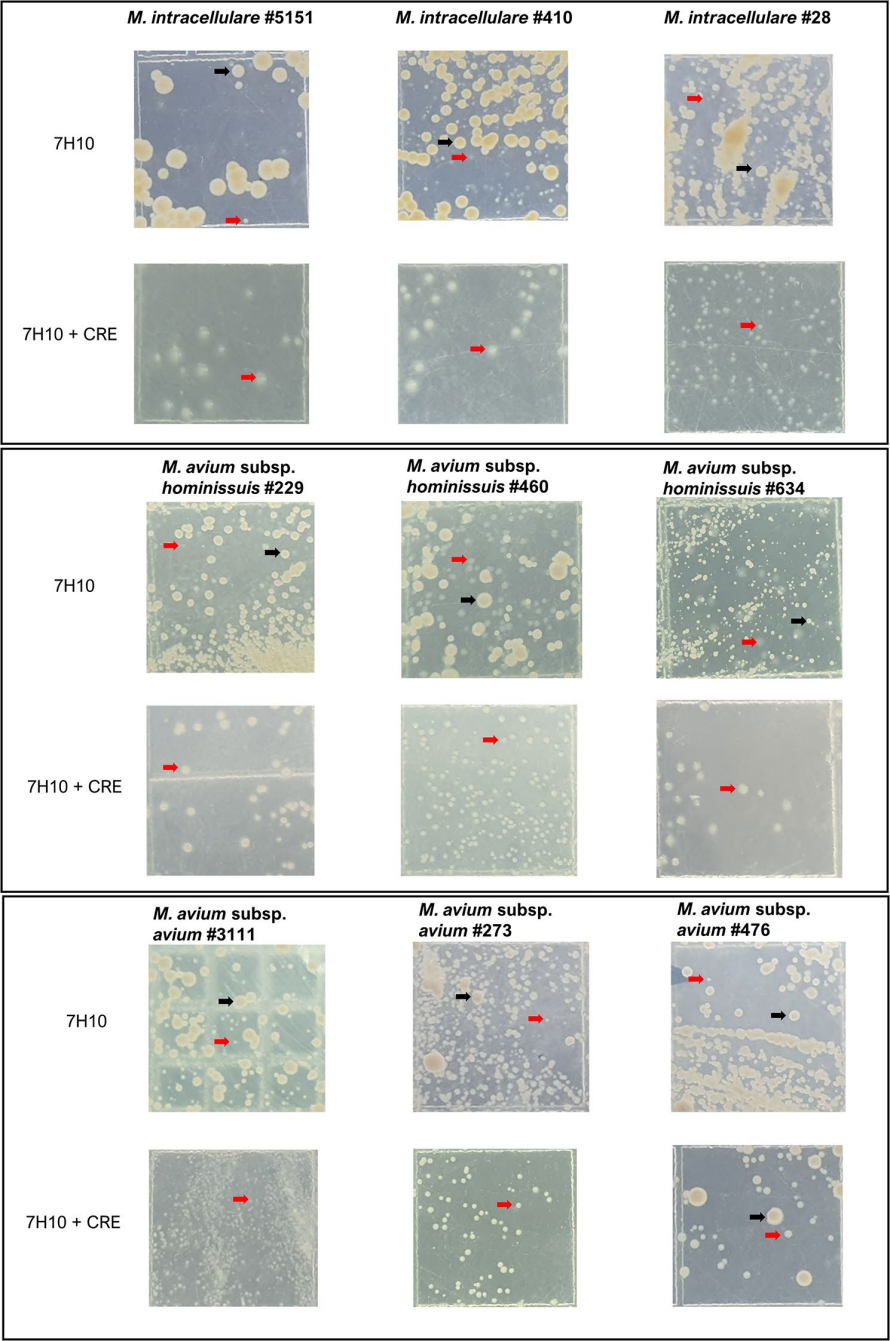
Initial observation of MAC SCVs phenotype from patients with MAC-pulmonary disease. Each primary stock of the MAC strain was inoculated onto both 7H10 agar and 7H10-CRE agar (containing clarithromycin at 2 µg/ml, rifampicin at 1 µg/ml, and ethambutol at 2 µg/ml) and incubated at 37 °C for 3 weeks. On 7H10 agar, the MAC strain exhibited a mixed colonial morphology, with dominant large and opaque colonies, as well as small, translucent colonies. In contrast, on 7H10-CRE agar, small, translucent colonies were predominant, except for the *M. avium* subsp. *avium* #476 strain, which displayed mixed colonial morphology. Large, opaque colonies are indicated by black arrows, and small, translucent colonies are indicated by red arrows

**Fig. 2:**
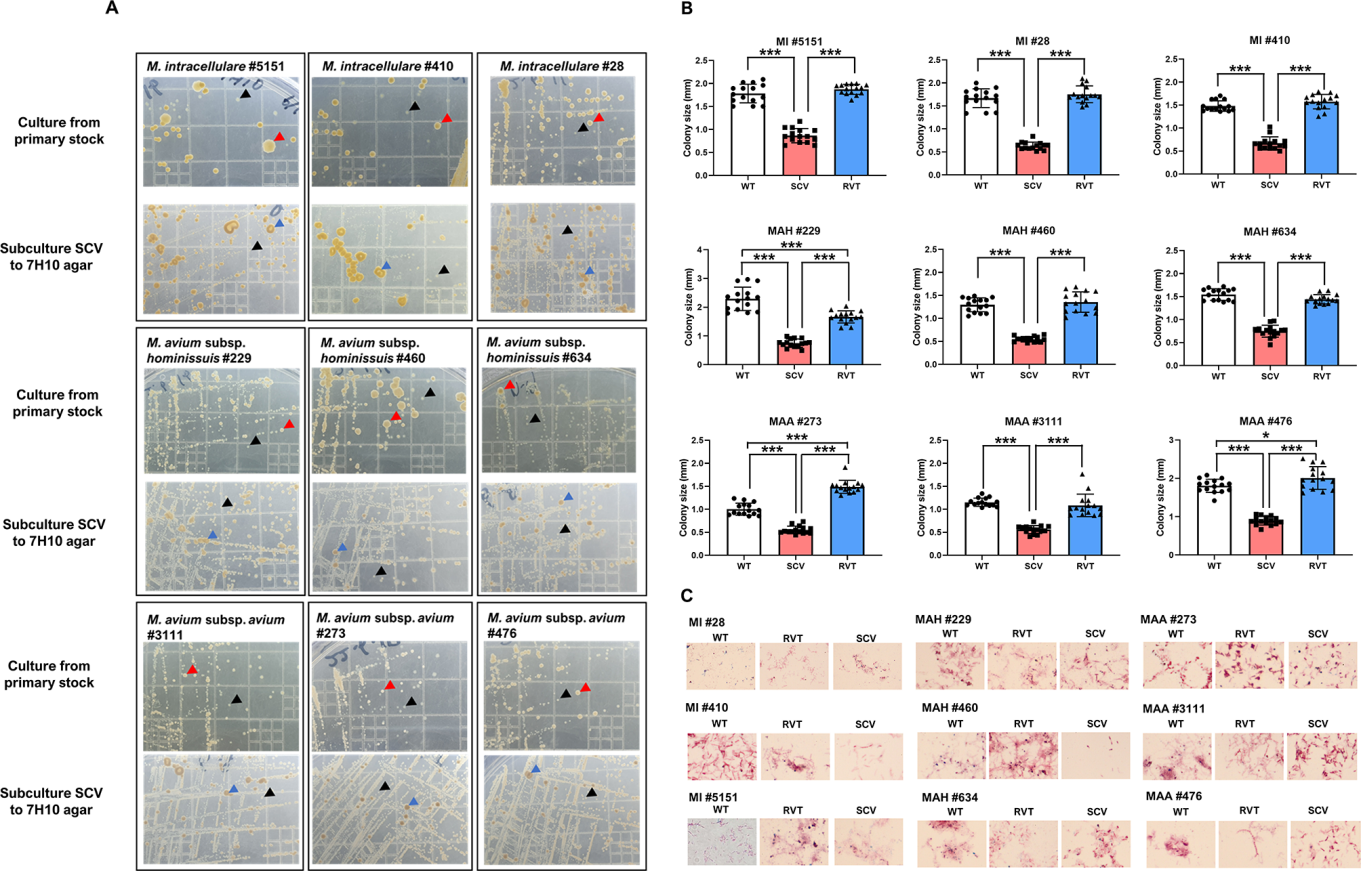
Colony morphology, size, and acid-fast staining results of WT, SCVs, and RVT MAC phenotypes from patients with MAC-pulmonary disease. (**A**) Colony morphology of wild type (WT), small colony variants (SCVs), and revertant (RVT) strains phenotypes. Two distinct colony morphotypes were observed on 7H10 agar after 4 weeks incubation at 37 °C. WT showed large, yellow-pigmented, and opaque colony morphology, while SCV demonstrated small and translucent colony in primary cultures. RVT phenotype was observed after the subculture of SCV to new 7H10 agar. RVT showed large, irregular, and opaque colony morphology. WT, SCVs, and RVT were marked with red, black, and blue arrowhead, respectively. (**B**) Colony size of WT, SCVs, and RVT strains was measured after 4 weeks of incubation at 37℃. SCVs showed a significantly smaller colony size than WT and RVT strains. During the incubation, the size of the SCVs colony was increased but did not exceed 1 mm in all MAC species. Error bars represent the standard deviation, and statistical significance was analyzed with the ANOVA (*, *p* < 0.05; **, *p* < 0.01; ***, *p* < 0.001). (**C**) Microscopic observation through the acid-fast staining of WT, SCV, and RVT strains. All WT, RVT, and SCT strains of MAC showed similar acid-fastness and bacterial cell morphology

Nine isolates (three per species), including both WT and SCV strains, were randomly selected from a pool of 49 MAC isolates. For *M. intracellulare*, MI #28, MI #410, and MI #5151 were chosen. In the case of *M. avium* subsp. *hominissuis*, MAH #229, MAH #460, and MAH #634 were selected. For *M. avium* subsp. *avium*, MAA #273, MAA #3111, and MAA #476 were chosen. During the initial screening, all MAC isolates containing SCVs displayed mixed colonies with varying morphologies, including large, opaque, small, and translucent colonies on 7H10 agar plates, as shown in Fig. 1. A standard combination of antimicrobial drugs for treatment, represented as CRE, reduced the large and opaque phenotype colonies but could not eliminate the SCV phenotypes, as presented in Fig. 1. Subsequently, after culturing on Middlebrook 7H10 agar, all SCVs exhibited small, gray to pale yellow colonies (Fig. 2A, indicated by a black arrowhead). In contrast, the WT strains displayed large, cream-to-yellow, opaque colonies (Fig. 2A, red arrowhead). Furthermore, WT-like colonies, represented as RVT, emerged from SCVs after prolonged incubation on Middlebrook 7H10 agar for 2–3 weeks (Fig. 2A, blue arrowhead). The SCV colonies were significantly smaller than the WT and RVT colonies in all MAC species (Fig. 2B). SCV colonies were observed after 2 weeks of incubation and grew during time-lapse but were still smaller than those of the WT and RVTs. The average colony size of SCV varied depending on the strains, from 0.54 to 0.89 mm. The average colony size of SCV did not exceed 1.0 mm after 4 weeks of incubation for any MAC species. We considered bacterial colonies with an average diameter of less than 1 mm that were, depigmented, and exhibited a smooth translucent gray appearance as SCVs (Fig. 2A). Acid-fast staining was performed for all strains to assess their acid-fastness and morphological characteristics. No significant differences in acid-fastness or morphology were observed between the WT, SCVs, and RVT strains by acid-fast staining (Fig. 2C).

### Growth curve

**Fig. 3:**
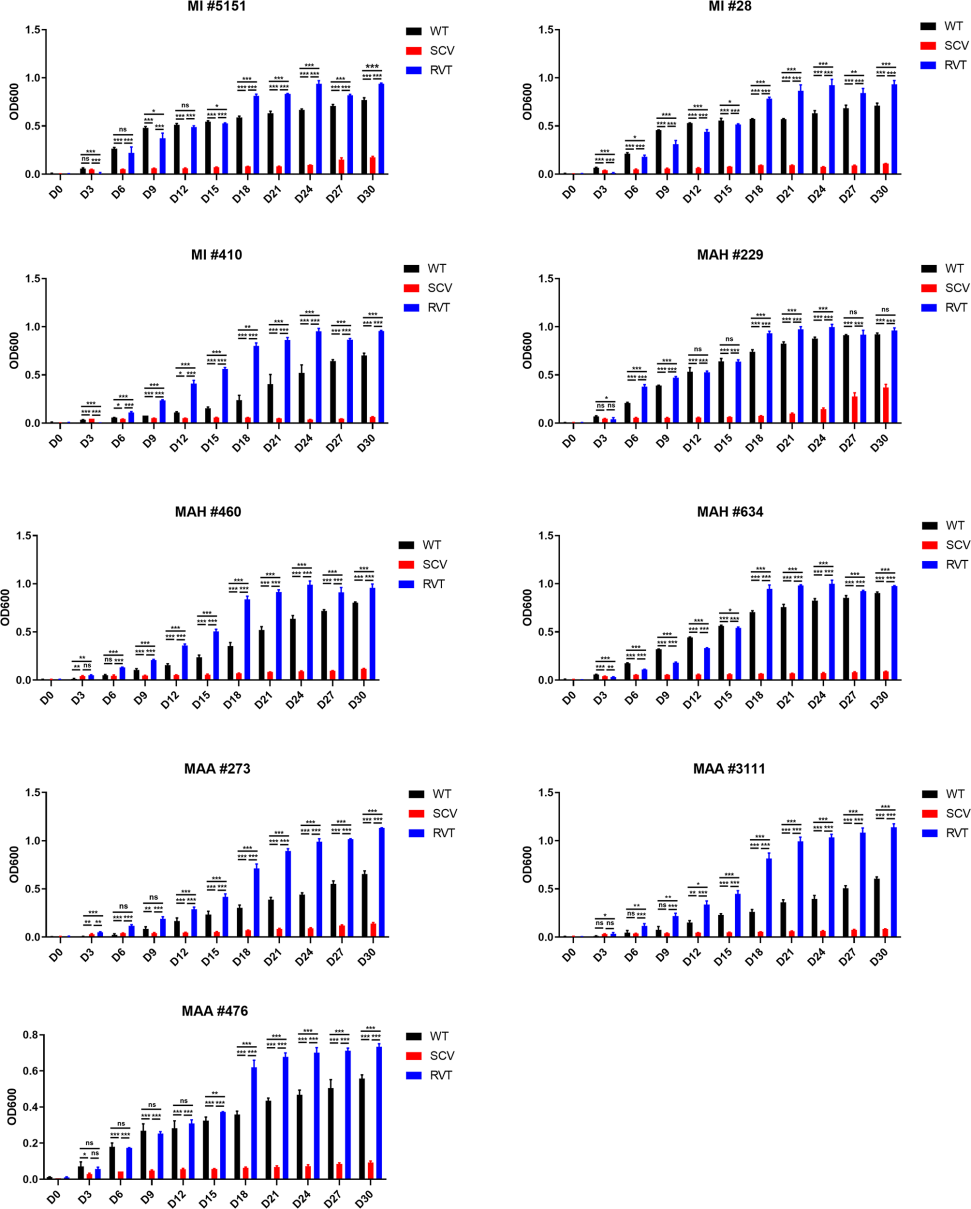
Growth curve of WT, SCVs, and RVT MAC phenotypes from patients with MAC-pulmonary disease. We measured the optical density of WT, SCV, and RVT phenotypes at 600 nm after the 30 days incubation. The SCVs showed a slower growth rate than the WT strain, whereas the RVT demonstrated a faster growth rate than the WT in 7H9 broth. All SCV exhibited a much longer lag phase than the WT and RVT phenotypes, and some strains remained in the lag phase for up to 30 days in 7H9 broth

In the growth curve analysis, the SCVs of MAC showed a slower growth rate than the WT strain, whereas the RVT strain displayed a faster growth rate in MB7H9 broth (Fig. 3). Briefly, all SCV strains showed a prolonged lag phase, and some strains did not grow for up to 30 days in MB7H9 broth (Fig. 3). For example, the SCVs strain of MI #410 showed an OD_600_ of 0.1 at 30 days. In addition, the SCVs strain of MAH #634 displayed an OD_600_ of0.08 at 30 days. Similarly, the SCVs strain of MAA #3111 and MAA #476 showed the OD_600_ of 0.08 and 0.09 at 30 days, respectively. In contrast, RVT strain displayed faster growth rates than the WT strain, as shown in Fig. 3. In detail, RVT strains of MI #410, MAH #460, MAA #3111, and MAA #273 exhibited faster growth rates than the WT strains.

### Molecular genotyping of MAC WT, SCVs, and RVT

In the molecular genotypic analysis, the WT, SCVs, and RVT strains isolated from the same patients showed identical VNTR profiles. The detailed VNTR profiles are listed in Table 2.

**Table 2:** VNTR profiles of wild-type, small colony variant, and revertant strains

*M. intracellulare* strains	VNTR locus															
	1	2	3	4	5	6	7	8	9	10	11	12	13	14	15	16
MI #5151 WT	2	1	1	1	2	3	1	2	1	1	4	2	2	2	1	2
MI #5151 SCV	2	1	1	1	2	3	1	2	1	1	4	2	2	2	1	2
MI #5151 RVT	2	1	1	1	2	3	1	2	1	1	4	2	2	2	1	2
MI #410 WT	2	1	1	2	2	3	2	2	2	4	2	2	3	2	2	2
MI #410 SCV	2	1	1	2	2	3	2	2	2	4	2	2	3	2	2	2
MI #410 RVT	2	1	1	2	2	3	2	2	2	4	2	2	3	2	2	2
MI #28 WT	2	3	2	1	2	3	2	2	2	5	2	2	2	2	2	2
MI #28 SCV	2	3	2	1	2	3	2	2	2	5	2	2	2	2	2	2
MI #28 RVT	2	3	2	1	2	3	2	2	2	5	2	2	2	2	2	2
***M. avium*** **strains**	**MATR locus**															
	**1**	**2**	**3**	**4**	**5**	**6**	**7**	**8**	**9**	**11**	**12**	**13**	**14**	**15**	**16**	
MAH #460 WT	2	2	4	1	3	1	3	1	1	5	3	2	4	4	2	
MAH #460 SCV	2	2	4	1	3	1	3	1	1	5	3	2	4	4	2	
MAH #460 RVT	2	2	4	1	3	1	3	1	1	5	3	2	4	4	2	
MAH #229 WT	2	0	1	2	2	1	5	1	1	1	3	0	2	2	2	
MAH #229 SCV	2	0	1	2	2	1	5	1	1	1	3	0	2	2	2	
MAH #229 RVT	2	0	1	2	2	1	5	1	1	1	3	0	2	2	2	
MAH #634 WT	2	2	2	2	3	3	3	3	1	5	3	2	4	4	1	
MAH #634 SCV	2	2	2	2	3	3	3	3	1	5	3	2	4	4	1	
MAH #634 RVT	2	2	2	2	3	3	3	3	1	5	3	2	4	4	1	
MAA #3111 WT	2	2	1	2	2	1	1	1	2	1	3	2	3	2	2	
MAA #3111 SCV	2	2	1	2	2	1	1	1	2	1	3	2	3	2	2	
MAA #3111 RVT	2	2	1	2	2	1	1	1	2	1	3	2	3	2	2	
MAA #273 WT	2	1	1	2	2	2	1	1	2	1	3	2	3	2	2	
MAA #273 SCV	2	1	1	2	2	2	1	1	2	1	3	2	3	2	2	
MAA #273 RVT	2	1	1	2	2	2	1	1	2	1	3	2	3	2	2	
MAA #476 WT	2	2	1	2	2	1	1	1	2	1	3	2	3	2	2	
MAA #476 SCV	2	2	1	2	2	1	1	1	2	1	3	2	3	2	2	
MAA #476 RVT	2	2	1	2	2	1	1	1	2	1	3	2	3	2	2	

### Comparison of MICs value among different phenotypes

**Fig. 4:**
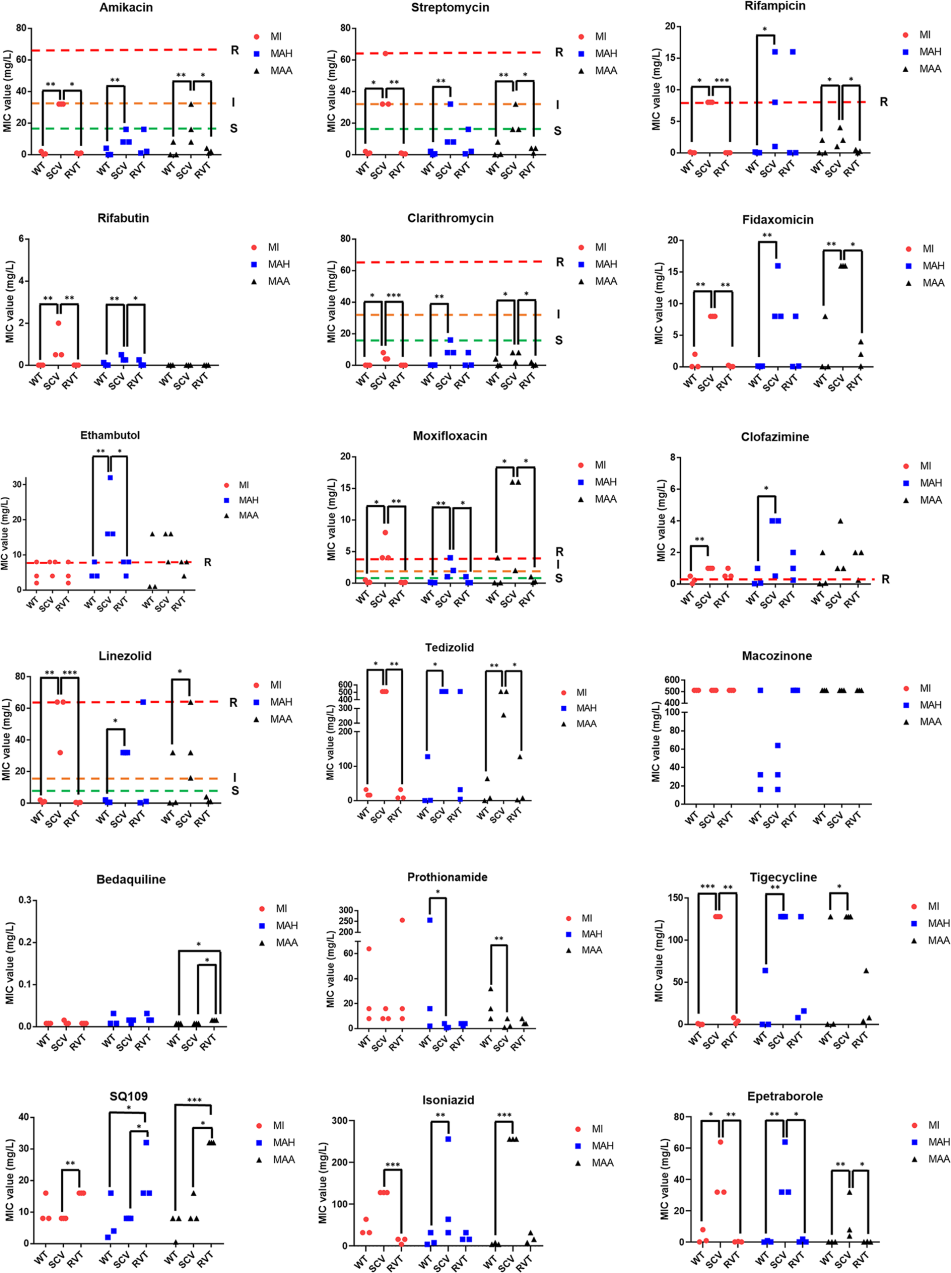
Comparison of MIC value between WT, SCVs, and RVT phenotypes. The MIC distributions of the MAC WT, SCVs, and RVT strains for 18 antimicrobial drugs are presented. Each dot represents the MIC for a single strain. The dashed lines represent the susceptibility breakpoints according to the CLSI and previous studies. Statistical significance was analyzed using the Kruskal-Wallis test (* *p* < 0.05; ** *p* < 0.01; *** *p* < 0.001)

A total of 18 antimicrobial drugs were tested for MIC determination against MAC WT, SCVs, and RVT strains. All SCVs showed higher MICs than the WT strain for most antimicrobial agents (Table 3). Briefly, SCVs displayed statistically significant augmentation in MICs for AMK, STR, RFP, RFB, CLR, FDX, MXF, CFZ, LZD, TZD, TGC, and EPB compared to the WT strain (Fig. 4). Moreover, the augmentation of MICs in SCVs compared to the WT varied depending on the MAC species. The SCVs of MI showed moderate augmentation of MICs for INZ compared to those of the WT strain, whereas MAA and MAH SCVs showed drastic augmentation of MICs, as shown in Table 3. Similarly, MI SCVs showed similar MICs for ETB, whereas MAA and MAH SCVs displayed moderate increases in MICs. In contrast, few antimicrobial drugs showed similar MICs for the WT, SCVs, and RVT strains. For example, the MICs of SQ109 were similar between the WT and SCVs, except for MAA #3111 and MAH #460. Also, MICs of MCZ were extremely high (512 µg/ml) in most strains. On the other hand, MICs of BDQ were considerably low (0.00783 to 0.03125 µg/ml) in all strains, including SCVs. Furthermore, some SCVs strains, such as MI #28, MAA #273, MAA #476, MAH #229, and MAH #460, showed lower MICs for PTM than the WT strain. The MIC values for the RVT strain were similar to those of the WT strain, with some exceptions. The RVT strain of MI #460 showed higher MICs for AMK, STR, CFZ, MCZ, SQ109, INZ, and TGC, whereas lower MICs for PTM. Similar patterns were observed in the RVT strains MAA #273, MAA #3111, and MAH #229. In contrast, the RVT strains MAA #476 and MAH #634 showed lower MICs for AMK, STR, RFP, FDX, MXF, LZD, TZD, and TGC. When we analyzed the clinical breakpoint, SCVs showed drastic augmentation of MICs over the breakpoint for some antibiotics, such as RFP, MXF, CFZ, and LZD, as shown in Fig. 4. In addition, the MICs of some drugs, such as AMK, STR, and CLR were increased in SCVs, but were still below the clinical breakpoints.

**Table 3:** MIC value distribution of wild-type, small colony variants, and revertant strains

Strains		Species	AMK	STR	RFP	RFB	ETB	CLR	FDX	MXF	CFZ	MCZ	BDQ	PTM	LZD	TZD	SQ109	TGC	INZ	EPB
#5151	WT	MI	0.5	1	≤ 0.0313	≤ 0.0078	2	≤ 0.0625	≤ 0.0625	0.125	≤ 0.0313	> 256	≤ 0.0078	8	0.5	16	8	≤ 0.0625	32	8
	SCV	MI	32	64	8	2	8	4	8	8	1	> 256	≤ 0.0078	8	64	> 256	8	> 64	128	32
	RVT	MI	1	0.5	≤ 0.0313	≤ 0.0078	4	≤ 0.0625	≤ 0.0625	≤ 0.0625	0.5	> 256	0.0078	8	0.5	8	16	1	16	0.25
#28	WT	MI	0.25	0.5	≤ 0.0313	≤ 0.0078	4	≤ 0.0625	≤ 0.0625	≤ 0.0625	0.25	> 256	≤ 0.0078	64	1	16	8	≤ 0.0625	32	0.25
	SCV	MI	32	32	8	0.5	4	8	8	4	1	> 256	0.0156	16	64	> 256	8	> 64	128	32
	RVT	MI	1	1	≤ 0.0313	0.0156	8	≤ 0.0625	0.25	0.125	1	> 256	0.0078	> 128	0.25	32	16	8	16	0.5
#410	WT	MI	2	2	0.125	≤ 0.0078	8	0.125	2	0.5	0.5	> 256	≤ 0.0078	16	2	32	16	1	64	1
	SCV	MI	32	32	8	0.5	8	4	8	4	1	> 256	≤ 0.0078	8	32	> 256	8	> 64	128	64
	RVT	MI	0.5	0.5	≤ 0.0313	≤ 0.0078	2	≤ 0.0625	≤ 0.0625	0.125	0.5	> 256	0.0078	16	0.5	8	16	4	4	0.25
#273	WT	MAA	≤ 0.125	≤ 0.125	≤ 0.0313	≤ 0.0078	1	≤ 0.0625	≤ 0.0625	≤ 0.0625	0.0625	> 256	≤ 0.0078	16	0.5	8	8	0.5	4	0.25
	SCV	MAA	8	16	1	≤ 0.0078	8	2	16	2	1	> 256	≤ 0.0078	2	16	256	8	> 64	> 256	4
	RVT	MAA	2	4	0.25	≤ 0.0078	8	0.25	4	0.25	2	> 256	0.0156	8	4	128	32	64	32	0.5
#476	WT	MAA	8	8	2	≤ 0.0078	16	4	8	4	2	> 256	≤ 0.0078	32	32	64	8	> 64	4	0.25
	SCV	MAA	16	16	4	≤ 0.0078	16	8	16	16	4	> 256	≤ 0.0078	1	64	> 256	16	> 64	> 256	8
	RVT	MAA	1	1	≤ 0.0313	≤ 0.0078	4	≤ 0.0625	0.125	0.125	2	> 256	0.0156	4	1	8	32	8	16	0.25
#3111	WT	MAA	≤ 0.125	0.25	≤ 0.0313	≤ 0.0078	1	≤ 0.0625	≤ 0.0625	≤ 0.0625	≤ 0.0313	> 256	≤ 0.0078	8	0.25	0.5	0.5	0.25	8	0.25
	SCV	MAA	32	32	2	≤ 0.0078	16	8	16	16	1	> 256	≤ 0.0078	8	32	> 256	8	> 64	> 256	32
	RVT	MAA	4	4	0.5	≤ 0.0078	8	2	2	1	0.25	> 256	0.0156	4	1	2	32	4	8	0.25
#229	WT	MAH	≤ 0.125	≤ 0.125	≤ 0.0313	≤ 0.0078	4	≤ 0.0625	≤ 0.0625	≤ 0.0625	≤ 0.0313	16	≤ 0.0078	16	0.5	0.5	4	≤ 0.0625	32	1
	SCV	MAH	8	8	16	0.5	16	8	16	4	4	16	0.0156	0.5	32	> 256	8	> 64	32	32
	RVT	MAH	16	16	16	0.25	8	8	16	1	2	> 256	0.0313	2	64	512	16	> 64	32	2
#460	WT	MAH	≤ 0.125	0.5	≤ 0.0313	≤ 0.0078	4	0.125	≤ 0.0625	≤ 0.0625	0.0625	32	≤ 0.0078	> 128	0.5	1	2	≤ 0.0625	4	≤ 0.125
	SCV	MAH	16	32	1	0.25	32	16	8	1	4	64	≤ 0.0078	4	32	> 256	8	> 64	> 256	64
	RVT	MAH	2	2	≤ 0.0313	≤ 0.0078	4	≤ 0.0625	≤ 0.0625	≤ 0.0625	1	> 256	0.0156	4	0.25	4	32	8	16	0.25
#634	WT	MAH	4	2	0.125	0.125	8	0.125	8	0.125	1	> 256	0.0313	2	2	128	16	64	8	0.25
	SCV	MAH	8	8	8	0.25	16	8	8	2	0.5	32	0.0156	1	32	> 256	8	> 64	64	32
	RVT	MAH	1	0.5	≤ 0.03125	≤ 0.0078	8	0.125	≤ 0.0625	≤ 0.0625	0.25	> 256	0.0156	4	1	32	16	16	16	0.25

The MIC values are presented up to four decimal places. Abbreviations: WT, Wild-type; SCV, Small colony-variants; RVT, Revertant; MI, *Mycobacterium intracellulare*; MAA, *Mycobacterium avium* subsp. *avium*; MAH, *Mycobacterium avium* subsp. *hominissuis*; AMK, Amikacin; STR, Streptomycin; RFP, Rifampicin; RFB, Rifabutin; ETB, Ethambutol; CLR, Clarithromycin; FDX, Fidaxomicin; MXF, Moxifloxacin; CFZ, Clofazimine; MCZ, Macozinone; BDQ, Bedaquiline; PTM, Prothionamide; LZD, Linezoild; TZD, Tedizolid; TGC, Tigecycline; INZ, Isoniazid; EPB, Epetraborole

Time-kill kinetics of three antimicrobial drug combinations against M. avium complex.

**Fig. 5:**
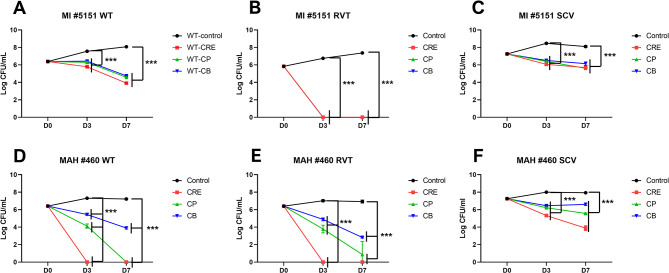
Time-kill kinetics of three antimicrobial drug combinations against *M. avium* complex. Two strains, *M. intracellulare* #5151 (**A** to **C**) and *M. avium* subsp. *hominissuis* #460 (**D** to **F**), were used in this analysis. The combinations included standard regimen drugs (CRE- containing clarithromycin at 2 µg/ml, rifampicin at 1 µg/ml, and ethambutol at 2 µg/ml), CB combination (containing clarithromycin at 2 µg/ml and bedaquiline at 0.25 µg/ml), and CP combination (containing clarithromycin at 2 µg/ml and prothionamide at 32 µg/ml). Error bars represent standard deviation, and statistical significance was analyzed using the ANOVA (* *p* < 0.05; ** *p* < 0.01; *** *p* < 0.001)

In the time-kill kinetics assay, all drug combinations significantly reduced the CFU on days 3 and 7 after treatment, as shown in Fig. 5. However, the extent of reduction varied depending on the phenotype and drug combination (Fig. 5). CRE was the most effective drug combination for MAC, followed by CP and CB. The RVT phenotype was the most susceptible to all drug combinations. No colonies grew on the MI #5151 RVT agar plates with any drug combination on days 3 and 7 (Fig. 5B). Similarly, when treated with the CRE combination, no colonies were observed on the MI #460 RVT agar plates on days 3 and 7 (Fig. 5E). The WT phenotype demonstrated intermediate susceptibility to all drug combinations (Fig. 5A and D). For instance, all drug combinations significantly reduced the CFU of MI #5151 WT on days 3 and 7; however, complete eradication was not achieved, as depicted in Fig. 5A. Additionally, the CRE combination eliminated MAH #460 WT on days 3 and 7 (Fig. 5D). The SCV phenotype exhibited slower killing rates than the WT and RVT phenotypes (Fig. 5C and F). While all drug combinations significantly reduced the CFU of SCV, none eliminated the bacteria (Fig. 5C and F).

## Discussion

Bacteria encounter a hostile environment with various stresses, such as oxidative stress, hyperosmolarity, nutrient starvation, hypoxia, pH alteration, and host immune response during infection within the host [32]. The phenotypic heterogeneity of bacteria that comprises small subpopulations within an isogenic strain can be an efficient survival strategy for persisting in a host-induced hostile environment [33, 34]. Persisters are a small subpopulation of bacteria that survive during antibiotic treatment without genetic mutation in the antibiotic targets [35]. Previous studies have shown that persisters can present as SCVs in clinical infections [18, 36, 37].

In the present study, we report for the first time the isolation of SCVs of the *M. avium* complex from patients with NTM lung disease. In total, 50 (46.7%) of 107 isolates of MAC were confirmed to have SCVs in their primary stock from Ogawa medium. These results align with those of previous studies that demonstrated that SCVs are frequently detected in patients with chronic bacterial infections [36, 38, 39]. The isolation rates of SCVs varied among studies [39–42]. Green et al. isolated *S. aureus* SCVs from 17 out of 260 cystic fibrosis patients [40]. In addition, Yagci et al. reported that 20 of 248 patients with cystic fibrosis harbored *S. aureus* SCVs [39]. Moreover, Wolter et al. isolated *S. aureus* SCVs from 24 out of 100 patients with cystic fibrosis [42]. More recently, Morelli et al. showed that *S. aureus* SCVs were recovered from 28 out of 222 patients with cystic fibrosis [41]. Compared to the study of SCVs of *S. aureus*, a high isolation rate of MAC SCVs was observed. Therefore, switching from a bacterial phenotype to a SCVs is expected in clinical MAC infections.

One of the unique characteristics of MAC SCVs is their slower growth rate compared to their WT counterparts. Several investigators have found slower growth rates of SCVs than of the WT strain in diverse bacterial pathogens, such as *Staphylococcus aureus*, *Pseudomonas aeruginosa*, *Escherichia coli*, *Stenotrophomonas maltophilia*, *Streptococcus tigurinus*, *Enterococcus faecalis*, *Salmonella enterica*, and *Ornithobacterium rhinotracheale* [43–51]. The slow growth rates of SCVs are associated with reduced metabolic activity, which has a beneficial effect on their persistence within the host. Metabolic regulation occurs in mycobacteria when they are exposed to hostile environments, such as hypoxia, acidic pH, nutrient starvation, and oxidative stress [52–54]. After these hostile conditions are alleviated, metabolic activity returns to normal, thereby promoting mycobacterial cell replication. This can, in turn, lead to the establishment of an active infection. The low metabolic states of mycobacteria enable them to evade the actions of antimicrobial drugs, that typically target actively growing cells [55]. Therefore, the metabolic state of bacteria affects their antibiotic susceptibility [56]. The stationary phase of bacteria, which resembles the physiological characteristics of SCVs, showed reduced metabolic activity and antibiotic tolerance to bactericidal antibiotics [56].

Clinically, enhanced antimicrobial resistance is the most relevant SCV phenotype associated with refractory or persistent infections. Previous studies reported that MICs of SCVs for various antibiotics are higher than the WT strain. Bogut et al. demonstrated that the MICs of gentamicin for SCV were 4 to 20-fold higher than those of the WT strains of *Staphylococcus epidermidis* and *Staphylococcus warneri* isolated from prosthetic joint infections [44]. Moreover, SCVs of *Stenotrophomonas maltophilia* isolated from sputum specimens of patients with cystic fibrosis had 4 to 128-fold and 1.5 to 2-fold higher MICs for levofloxacin and sulfamethoxazole [43]. Recently, Millette et al. reported that SCVs of *Staphylococcus aureus* isolated from patients with cystic fibrosis showed 2 to 64-fold higher MICs for gentamicin and tobramycin [57]. Consistently, a substantial increase in the MICs was observed in MAC SCVs against multiple antibiotics in the present study.

Conventional antimicrobial drugs target bacterial cellular processes, such as ATP biosynthesis, cell wall biogenesis, DNA replication, transcription, and protein synthesis [58]. Cellular processes targeted by antibiotics involve cell growth and division and require considerable energy. Conventional antibiotics are effective against metabolically active bacterial cells [59]. The antimicrobial drugs that showed statistically significant augmentation of MICs in MAC SCVs compared to the WT were those targeting metabolically active cells. For example, aminoglycosides and tetracyclines bind to the 30 S subunit of the bacterial ribosome and induce mistranslation of proteins [58]. Additionally, macrolides and oxazolidinones inhibit the 50 S subunit of bacterial ribosome [56]. Similarly, EPB binds to the editing domain of leucyl-tRNA synthetase, subsequently blocking the protein synthesis [60]. Fast-growing bacterial cells are more susceptible to ribosome-targeting antibiotics [61]. Therefore, the slow growth rate of SCVs may result in low levels of protein synthesis, thereby increasing tolerance to antibiotics that target protein synthesis.

Antimicrobial drugs that target nucleic acid synthesis and bacterial respiration depend on their metabolic activity. The slow or arrested growth of SCV, which represents low rates of DNA and RNA synthesis, confers considerable antibiotic tolerance to rifamycin (RFP and RFB), quinolone (MXF), and CFZ. Further investigations are required to restore the metabolic activity of MAC SCV and augment the effects of drugs. The resuscitation-promoting factors (Rpfs) are secreted signaling molecules that modulate the growth and replication of dormant bacteria [62]. Previous studies have demonstrated that Rpfs from *Micrococcus luteus* can stimulate arrested bacterial growth, thereby improving the cultivation of environmental bacteria [63, 64]. The supplementation of Rpfs may stimulate the growth of MAC SCV and enhance the killing effect of conventional antimicrobial drugs.

On the contrary, some antibiotics had similar or lower MICs in the SCVs than in the WT. For example, BDQ showed extremely low MICs in all phenotypes. BDQ is a newly developed diarylquinoline that specifically inhibits bacterial ATP-synthase [65]. Although ATP production is strongly associated with bacterial metabolic activity, it is unclear why the MICs of BDQ are low in MAC SCVs. Further analysis, such as the quantification of ATP production among the WT, SCVs, and RVT, is needed to prove this mystery. Similarly, PTM showed similar or lower MICs in SCVs than in the WT. PTM are thioamides and has been used to treat drug-resistant tuberculosis [66]. As a prodrug, PTM produces covalent adducts with nicotinamide adenine dinucleotide and inhibits fatty acid synthesis by binding to inhA [66]. Pathogenic mycobacteria use a variety of carbon sources [67]. Host-derived fatty acids are important carbon sources for mycobacteria inside host macrophages [68–70]. Previous studies have reported that fatty acid pathways are enriched in host-mimicking environments, including nutrient starvation and biofilm conditions, and are associated with antibiotic susceptibility [71, 72]. Thus, inhibition of fatty acid synthesis by PTM may effectively eliminate the SCV phenotype.

In the killing kinetics analysis, none of the drug combinations, including BDQ and PTM, sterilized the SCV phenotype. In contrast, RVT was readily sterilized by CRE drug combinations for MAH and CRE, CP, and CB combinations for MI. RVT has a higher growth rate than SCV; therefore, metabolic activity is expected to be restored after the transition from SCV to RVT. Typically, anti-TB drugs are effective against actively replicating mycobacteria [73], which explain why all drug combinations are effective against RVT. Therefore, identifying the activated metabolism in each phenotype will lead to finding ways to enhance the effectiveness of current treatment regimen in further research.

Developing heterogeneous bacterial populations within the host is a suitable survival strategy that confers fitness to mycobacteria to persist during infection [74–76]. Previous studies have demonstrated that the rapid correction of frameshift mutation of *M. tuberculosis* provides phenotypic switching to enhance persistence within the host [77, 78]. Safi et al. revealed that a frameshift mutation of the *glpK* gene confers phenotypic switching to a small and smooth colony phenotype from *M. tuberculosis* clinical strains [77]. The small colony phenotype shows slow growth and reduced susceptibility to various antituberculosis drugs [77]. Furthermore, small colony phenotypes emerged during murine infection by acquiring frameshift mutation in the *glpK* gene [77]. More recently, Safi et al. identified that a frameshift mutation of the *orn* gene result in a small colony phenotype and ethambutol resistance in *M. tuberculosis* [78]. Rapid reversion of the frameshift of the *orn* gene restores the ethambutol MIC and colony phenotype to those of the WT [78]. These studies suggest that reversible frameshift mutations contribute to the fitness of *M. tuberculosis* for adaptation during infection.

The present study observed a similar small colony phenotype of MAC in patients with NTM-lung disease. MAC SCV displayed characteristics similar to those of the *M. tuberculosis* small colony phenotype investigated in previous studies. For example, SCV showed slower growth rates than the WT and could revert to a WT-like phenotype. Moreover, the MICs of SCV were much higher than those of the WT and showed reduced susceptibility during the killing kinetics analysis. However, we found distinct differences in MAC SCV compared with small colonies of *M. tuberculosis*. First, MAC SCV did not entirely revert to WT-like colonies, and WT-like colonies grew from the SCV after prolonged incubation. Second, MAC SCV exhibited much higher MICs (up to 2048-fold) than the WT and RVT, whereas the *M. tuberculosis* small colony phenotype showed similar or 2-fold higher MICs. These observations suggest that the MAC SCV phenotype involves mechanisms different from those of *M. tuberculosis*. Unfortunately, we did not analyze single nucleotide polymorphisms or transcriptional changes between different the MAC phenotypes. Thus, whole-genome and transcriptional analyses are necessary to elucidate phenotype-switching mechanisms in MAC.

Our study had several limitations. First, the variables investigated from electronic medical records may be inaccurate or missed. For example, only a few patients diagnosed with MAC lung disease have bronchiectasis. Second, we did not obtain radiological findings, clinical symptoms, treatment duration, or pulmonary function data. Revealing the relationship between the emergence of SCV, radiographic changes, and treatment duration strengthens the clinical relevance. Furthermore, we could not confirm whether patients fulfilled the ATS/IDSA diagnostic criteria for MAC lung disease [79]. Thus, it was challenging to elucidate the microbiological status, such as specimen contamination, transient colonization, and true infection, between patients in the present study. Moreover, our findings were limited by the fact that the data were single-institutional, as well as the retrospective characteristics of the study. Further research is required to explore the association of SCV emergence and clinical outcomes using accurate patient data and a larger cohort from multiple centers.

## Conclusion

In conclusion, this study elucidated the phenotypic traits of SCVs in MAC, thereby enhancing our understanding of MAC’s life cycle for persistence within the host. These SCVs within the MAC can revert to a WT-like phenotype known as RVT. Furthermore, their reduced metabolic activity and increased antibiotic resistance may serve as survival strategies to overcome the challenging host environment during infection. Based on our findings, two potential approaches can be considered to enhance the treatment of MAC infections, including those involving SCVs. First, activation of the conversion of SCVs to RVT may be an effective treatment strategy. Second, combining current therapeutic drugs with potential drugs targeting SCVs may be a promising strategy for improving clinical outcomes.

## Data Availability

All data produced or investigated during this study are included in this published article.

## References

[1] Koh WJ. Nontuberculous Mycobacteria-Overview. Microbiol Spectr. 2017;5(1). 10.1128/microbiolspec.TNMI7-0024-2016. 10.1128/microbiolspec.tnmi7-0024-2016 PMC11687458 28128073

[2] Honda JR, Virdi R, Chan ED (2018). Global Environmental Nontuberculous Mycobacteria and their contemporaneous man-made and natural niches. Front Microbiol.

[3] Henkle E, Winthrop KL (2015). Nontuberculous mycobacteria infections in immunosuppressed hosts. Clin Chest Med.

[4] Daley CL. Mycobacterium avium Complex Disease. Microbiol Spectr. 2017;5(2). 10.1128/microbiolspec.TNMI7-0045-2017. 10.1128/microbiolspec.tnmi7-0045-2017 PMC11687487 28429679

[5] Riccardi N, Monticelli J, Antonello RM, Luzzati R, Gabrielli M, Ferrarese M (2020). Mycobacterium chimaera infections: an update. J Infect Chemother.

[6] Boonjetsadaruhk W, Kaewprasert O, Nithichanon A, Ananta P, Chaimanee P, Salao K (2022). High rate of reinfection and possible transmission of Mycobacterium avium complex in Northeast Thailand. One Health.

[7] Boyle DP, Zembower TR, Qi C (2016). Relapse versus Reinfection of Mycobacterium avium Complex Pulmonary Disease. Patient characteristics and Macrolide susceptibility. Ann Am Thorac Soc.

[8] Kwon YS, Koh WJ, Daley CL (2019). Treatment of Mycobacterium avium Complex Pulmonary Disease. Tuberc Respir Dis (Seoul).

[9] Jeong BH, Jeon K, Park HY, Kim SY, Lee KS, Huh HJ (2015). Intermittent antibiotic therapy for nodular bronchiectatic Mycobacterium avium complex lung disease. Am J Respir Crit Care Med.

[10] Koh WJ, Moon SM, Kim SY, Woo MA, Kim S, Jhun BW, et al. Outcomes of Mycobacterium avium complex lung disease based on clinical phenotype. Eur Respir J. 2017;50(3). 10.1183/13993003.02503-2016. 10.1183/13993003.02503-2016 28954780

[11] Kwak N, Park J, Kim E, Lee CH, Han SK, Yim JJ (2017). Treatment outcomes of Mycobacterium avium Complex Lung Disease: a systematic review and Meta-analysis. Clin Infect Dis.

[12] Jhun BW, Moon SM, Kim SY, Park HY, Jeon K, Kwon OJ, et al. Intermittent antibiotic therapy for recurrent nodular bronchiectatic Mycobacterium avium Complex Lung Disease. Antimicrob Agents Chemother. 2018;62(2). 10.1128/AAC.01812-17. 10.1128/AAC.01812-17 PMC5786774 29203483

[13] Kwon BS, Shim TS, Jo KW. The second recurrence of Mycobacterium avium complex lung disease after successful treatment for first recurrence. Eur Respir J. 2019;53(1). 10.1183/13993003.01038-2018. 10.1183/13993003.01038-2018 30337449

[14] Lee BY, Kim S, Hong Y, Lee SD, Kim WS, Kim DS (2015). Risk factors for recurrence after successful treatment of Mycobacterium avium complex lung disease. Antimicrob Agents Chemother.

[15] Besse A, Groleau MC, Deziel E (2023). Emergence of small colony variants is an adaptive strategy used by Pseudomonas aeruginosa to mitigate the effects of Redox Imbalance. mSphere.

[16] Besse A, Groleau MC, Trottier M, Vincent AT, Deziel E (2022). Pseudomonas aeruginosa strains from both clinical and environmental origins readily adopt a stable small-colony-variant phenotype resulting from single mutations in c-di-GMP pathways. J Bacteriol.

[17] Bollar GE, Keith JD, Oden AM, Kiedrowski MR, Birket SE (2022). Acute infection with a Tobramycin-Induced small colony variant of Staphylococcus aureus causes increased inflammation in the cystic fibrosis rat lung. Infect Immun.

[18] Fauerharmel-Nunes T, Flannagan RS, Goncheva MI, Bayer AS, Fowler VG, Chan LC (2023). MRSA isolates from patients with persistent bacteremia Generate Nonstable small colony variants in Vitro within macrophages and endothelial cells during prolonged vancomycin exposure. Infect Immun.

[19] Liu S, Chen H, Chen J, Wang T, Tu S, Zhang X (2023). Transcriptome and proteome of Methicillin-Resistant Staphylococcus aureus small-colony variants reveal changed metabolism and increased Immune Evasion. Microbiol Spectr.

[20] Proctor RA, von Eiff C, Kahl BC, Becker K, McNamara P, Herrmann M (2006). Small colony variants: a pathogenic form of bacteria that facilitates persistent and recurrent infections. Nat Rev Microbiol.

[21] Kahl BC, Becker K, Loffler B (2016). Clinical significance and Pathogenesis of Staphylococcal small colony variants in persistent infections. Clin Microbiol Rev.

[22] Li W, Li Y, Wu Y, Cui Y, Liu Y, Shi X (2016). Phenotypic and genetic changes in the life cycle of small colony variants of Salmonella enterica serotype typhimurium induced by streptomycin. Ann Clin Microbiol Antimicrob.

[23] McCarthy C (1970). Spontaneous and Induced Mutation in Mycobacterium avium. Infect Immun.

[24] Schaefer WB, Davis CL, Cohn ML (1970). Pathogenicity of transparent, opaque, and rough variants of Mycobacterium avium in chickens and mice. Am Rev Respir Dis.

[25] Stormer RS, Falkinham JO (1989). Differences in antimicrobial susceptibility of pigmented and unpigmented colonial variants of Mycobacterium avium. J Clin Microbiol.

[26] Kim MJ, Kim KM, Shin JI, Ha JH, Lee DH, Choi JG (2021). Identification of Nontuberculous Mycobacteria in patients with Pulmonary diseases in Gyeongnam, Korea, using multiplex PCR and Multigene Sequence-Based Analysis. Can J Infect Dis Med Microbiol.

[27] Woods GL, Brown-Elliott BA, Conville PS, Desmond EP, Hall GS, Lin G, et al. Susceptibility testing of Mycobacteria, Nocardiae, and other Aerobic actinomycetes. Wayne (PA); 2011. 31339680

[28] Brown-Elliott BA, Woods GL. Antimycobacterial susceptibility testing of Nontuberculous Mycobacteria. J Clin Microbiol. 2019;57(10). 10.1128/JCM.00834-19. 10.1128/JCM.00834-19 PMC6760954 31315954

[29] Kwak N, Whang J, Yang JS, Kim TS, Kim SA, Yim JJ (2021). Minimal inhibitory concentration of Clofazimine among Clinical isolates of Nontuberculous Mycobacteria and its impact on treatment outcome. Chest.

[30] Ichikawa K, Yagi T, Inagaki T, Moriyama M, Nakagawa T, Uchiya KI (2010). Molecular typing of Mycobacterium intracellulare using multilocus variable-number of tandem-repeat analysis: identification of loci and analysis of clinical isolates. Microbiol (Reading).

[31] Inagaki T, Nishimori K, Yagi T, Ichikawa K, Moriyama M, Nakagawa T (2009). Comparison of a variable-number tandem-repeat (VNTR) method for typing Mycobacterium avium with mycobacterial interspersed repetitive-unit-VNTR and IS1245 restriction fragment length polymorphism typing. J Clin Microbiol.

[32] Fang FC, Frawley ER, Tapscott T, Vazquez-Torres A (2016). Bacterial stress responses during host infection. Cell Host Microbe.

[33] Reyes Ruiz LM, Williams CL, Tamayo R (2020). Enhancing bacterial survival through phenotypic heterogeneity. PLoS Pathog.

[34] Weigel WA, Dersch P (2018). Phenotypic heterogeneity: a bacterial virulence strategy. Microbes Infect.

[35] Fisher RA, Gollan B, Helaine S (2017). Persistent bacterial infections and persister cells. Nat Rev Microbiol.

[36] Huemer M, Mairpady Shambat S, Bergada-Pijuan J, Soderholm S, Boumasmoud M, Vulin C, et al. Molecular reprogramming and phenotype switching in Staphylococcus aureus lead to high antibiotic persistence and affect therapy success. Proc Natl Acad Sci U S A. 2021;118(7). 10.1073/pnas.2014920118. 10.1073/pnas.2014920118 PMC7896289 33574060

[37] Vulin C, Leimer N, Huemer M, Ackermann M, Zinkernagel AS (2018). Prolonged bacterial lag time results in small colony variants that represent a sub-population of persisters. Nat Commun.

[38] Sendi P, Frei R, Maurer TB, Trampuz A, Zimmerli W, Graber P (2010). Escherichia coli variants in periprosthetic joint infection: diagnostic challenges with sessile bacteria and sonication. J Clin Microbiol.

[39] Yagci S, Hascelik G, Dogru D, Ozcelik U, Sener B (2013). Prevalence and genetic diversity of Staphylococcus aureus small-colony variants in cystic fibrosis patients. Clin Microbiol Infect.

[40] Green N, Burns JL, Mayer-Hamblett N, Kloster M, Lands LC, Anstead M (2011). Lack of association of small-colony-variant Staphylococcus aureus strains with long-term use of azithromycin in patients with cystic fibrosis. J Clin Microbiol.

[41] Morelli P, De Alessandri A, Manno G, Marchese A, Bassi M, Lobello R (2015). Characterization of Staphylococcus aureus small colony variant strains isolated from Italian patients attending a regional cystic fibrosis care centre. New Microbiol.

[42] Wolter DJ, Emerson JC, McNamara S, Buccat AM, Qin X, Cochrane E (2013). Staphylococcus aureus small-colony variants are independently associated with worse lung disease in children with cystic fibrosis. Clin Infect Dis.

[43] Anderson SW, Stapp JR, Burns JL, Qin X (2007). Characterization of small-colony-variant Stenotrophomonas maltophilia isolated from the sputum specimens of five patients with cystic fibrosis. J Clin Microbiol.

[44] Bogut A, Niedzwiadek J, Koziol-Montewka M, Strzelec-Nowak D, Blacha J, Mazurkiewicz T (2014). Characterization of Staphylococcus epidermidis and staphyloccocus warneri small-colony variants associated with prosthetic-joint infections. J Med Microbiol.

[45] Gomez-Gonzalez C, Acosta J, Villa J, Barrado L, Sanz F, Orellana MA (2010). Clinical and molecular characteristics of infections with CO2-dependent small-colony variants of Staphylococcus aureus. J Clin Microbiol.

[46] Masoud-Landgraf L, Zarfel G, Kaschnigg T, Friedl S, Feierl G, Wagner-Eibel U (2016). Analysis and characterization of Staphylococcus aureus small colony variants isolated from cystic fibrosis patients in Austria. Curr Microbiol.

[47] Petersen A, Chadfield MS, Christensen JP, Christensen H, Bisgaard M (2008). Characterization of small-colony variants of Enterococcus faecalis isolated from chickens with amyloid arthropathy. J Clin Microbiol.

[48] Roggenkamp A, Sing A, Hornef M, Brunner U, Autenrieth IB, Heesemann J (1998). Chronic prosthetic hip infection caused by a small-colony variant of Escherichia coli. J Clin Microbiol.

[49] Wellinghausen N, Chatterjee I, Berger A, Niederfuehr A, Proctor RA, Kahl BC (2009). Characterization of clinical Enterococcus faecalis small-colony variants. J Clin Microbiol.

[50] Zahra M, Ferreri M, Alkasir R, Yin J, Han B, Su J (2013). Isolation and characterization of small-colony variants of Ornithobacterium rhinotracheale. J Clin Microbiol.

[51] Zbinden A, Quiblier C, Hernandez D, Herzog K, Bodler P, Senn MM (2014). Characterization of Streptococcus Tigurinus small-colony variants causing prosthetic joint infection by comparative whole-genome analyses. J Clin Microbiol.

[52] Baruzzo G, Serafini A, Finotello F, Sanavia T, Cioetto-Mazzabo L, Boldrin F (2023). Role of the extracytoplasmic function sigma factor SigE in the stringent response of Mycobacterium tuberculosis. Microbiol Spectr.

[53] Choudhary E, Sharma R, Pal P, Agarwal N (2022). Deciphering the Proteomic Landscape of Mycobacterium tuberculosis in response to Acid and oxidative stresses. ACS Omega.

[54] Singh PR, Vijjamarri AK, Sarkar D. Metabolic switching of Mycobacterium tuberculosis during Hypoxia is controlled by the Virulence Regulator PhoP. J Bacteriol. 2020;202(7). 10.1128/JB.00705-19. 10.1128/JB.00705-19 PMC7167471 31932312

[55] Gomez JE, McKinney JD (2004). M. Tuberculosis persistence, latency, and drug tolerance. Tuberculosis (Edinb).

[56] Stokes JM, Lopatkin AJ, Lobritz MA, Collins JJ (2019). Bacterial metabolism and antibiotic efficacy. Cell Metab.

[57] Millette G, Seguin DL, Isabelle C, Chamberland S, Lucier JF, Rodrigue S, et al. Staphylococcus aureus small-colony variants from airways of adult cystic fibrosis patients as precursors of adaptive antibiotic-resistant mutations. Antibiot (Basel). 2023;12(6). 10.3390/antibiotics12061069. 10.3390/antibiotics12061069 PMC10294822 37370388

[58] Kohanski MA, Dwyer DJ, Collins JJ (2010). How antibiotics kill bacteria: from targets to networks. Nat Rev Microbiol.

[59] Lobritz MA, Belenky P, Porter CB, Gutierrez A, Yang JH, Schwarz EG (2015). Antibiotic efficacy is linked to bacterial cellular respiration. Proc Natl Acad Sci U S A.

[60] Baker SJ, Zhang YK, Akama T, Lau A, Zhou H, Hernandez V (2006). Discovery of a new boron-containing antifungal agent, 5-fluoro-1,3-dihydro-1-hydroxy-2,1- benzoxaborole (AN2690), for the potential treatment of onychomycosis. J Med Chem.

[61] Greulich P, Scott M, Evans MR, Allen RJ (2015). Growth-dependent bacterial susceptibility to ribosome-targeting antibiotics. Mol Syst Biol.

[62] Ravagnani A, Finan CL, Young M (2005). A novel firmicute protein family related to the actinobacterial resuscitation-promoting factors by non-orthologous domain displacement. BMC Genomics.

[63] Guzman J, Raval D, Hauck D, Titz A, Poehlein A, Degenkolb T, et al. The resuscitation-promoting factor (rpf) from Micrococcus luteus and its putative reaction product 1,6-anhydro-MurNAc increase culturability of environmental bacteria. Access Microbiol. 2023;5(9). 10.1099/acmi.0.000647.v4. 10.1099/acmi.0.000647.v4 PMC10569661 37841103

[64] Lopez Marin MA, Strejcek M, Junkova P, Suman J, Santrucek J, Uhlik O (2021). Exploring the potential of Micrococcus luteus Culture Supernatant with resuscitation-promoting factor for enhancing the culturability of soil Bacteria. Front Microbiol.

[65] Diacon AH, Pym A, Grobusch MP, de los Rios JM, Gotuzzo E, Vasilyeva I (2014). Multidrug-resistant tuberculosis and culture conversion with bedaquiline. N Engl J Med.

[66] Wang F, Langley R, Gulten G, Dover LG, Besra GS, Jacobs WR (2007). Mechanism of thioamide drug action against tuberculosis and leprosy. J Exp Med.

[67] de Carvalho LP, Fischer SM, Marrero J, Nathan C, Ehrt S, Rhee KY (2010). Metabolomics of Mycobacterium tuberculosis reveals compartmentalized co-catabolism of carbon substrates. Chem Biol.

[68] Kim MJ, Wainwright HC, Locketz M, Bekker LG, Walther GB, Dittrich C (2010). Caseation of human tuberculosis granulomas correlates with elevated host lipid metabolism. EMBO Mol Med.

[69] Lee W, VanderVen BC, Fahey RJ, Russell DG (2013). Intracellular Mycobacterium tuberculosis exploits host-derived fatty acids to limit metabolic stress. J Biol Chem.

[70] Prakhar P, Bhatt B, Lohia GK, Shah A, Mukherjee T, Kolthur-Seetharam U (2023). G9a and Sirtuin6 epigenetically modulate host cholesterol accumulation to facilitate mycobacterial survival. PLoS Pathog.

[71] Abukhalid N, Rojony R, Danelishvili L, Bermudez LE (2023). Metabolic pathways that permit Mycobacterium avium subsp. hominissuis to transition to different environments encountered within the host during infection. Front Cell Infect Microbiol.

[72] Silva C, Rojony R, Bermudez LE, Danelishvili L. Short-chain fatty acids promote Mycobacterium avium subsp. hominissuis growth in Nutrient-Limited environments and influence susceptibility to antibiotics. Pathogens. 2020;9(9). 10.3390/pathogens9090700. 10.3390/pathogens9090700 PMC7559849 32859077

[73] Baek SH, Li AH, Sassetti CM (2011). Metabolic regulation of mycobacterial growth and antibiotic sensitivity. PLoS Biol.

[74] Born SEM, Reichlen MJ, Bartek IL, Benoit JB, Frank DN, Voskuil MI. Population heterogeneity in Mycobacterium smegmatis and Mycobacterium abscessus. Microbiol (Reading). 2023;169(10). 10.1099/mic.0.001402. 10.1099/mic.0.001402 PMC10634367 37862100

[75] Chung ES, Johnson WC, Aldridge BB (2022). Types and functions of heterogeneity in mycobacteria. Nat Rev Microbiol.

[76] Walter ND, Ernest JP, Dide-Agossou C, Bauman AA, Ramey ME, Rossmassler K (2023). Lung microenvironments harbor Mycobacterium tuberculosis phenotypes with distinct treatment responses. Antimicrob Agents Chemother.

[77] Safi H, Gopal P, Lingaraju S, Ma S, Levine C, Dartois V (2019). Phase variation in Mycobacterium tuberculosis glpK produces transiently heritable drug tolerance. Proc Natl Acad Sci U S A.

[78] Safi H, Lingaraju S, Ma S, Husain S, Hoque M, Soteropoulos P, et al. Rapidly correcting frameshift mutations in the Mycobacterium tuberculosis Orn Gene produce reversible Ethambutol Resistance and small-colony-variant morphology. Antimicrob Agents Chemother. 2020;64(9). 10.1128/AAC.00213-20. 10.1128/AAC.00213-20 PMC7449195 32571828

[79] Daley CL, Iaccarino JM, Lange C, Cambau E, Wallace RJ, Andrejak C (2020). Treatment of Nontuberculous Mycobacterial Pulmonary Disease: an Official ATS/ERS/ESCMID/IDSA Clinical Practice Guideline. Clin Infect Dis.

